# Michigan Dental Association

**Published:** 1864-02

**Authors:** 


### Proceedings of Societies.

### MICHIGAN DENTAL ASSOCIATION.

JACKSON, Jan. 19th, 1864.

The ninth annual session met, pursuant to adjournment, at the "Hibbard House." President, Dr. Benedict, in the Chair. Minutes of the last meeting read and approved.

Drs. Porter, Stone and Whiting were appointed a committee to examine candidates for membership.

The following named persons were examined and admitted: Drs. Thomas Rix of Clinton, D. A. Wilder and G. H. Mosher of Jackson, H. Cole of Grand Ledge, E. Hawes of Tecumseh, and D. W. Stone of Jonesville.

The following officers were chosen for the ensuing year: Dr. G. W. STONE, of Albion, President; Dr. J. A. WATLING, of Ypsilanti, Vice-President; Dr. G. H. MOSHER, of Jackson, Recording & Corresponding Secretary; Dr. C. B. PORTER, of Ann Arbor, Treasurer; Drs. J. A. ROBINSON, H. BENEDICT and S. C. WHITING, Examining, Executive and Publishing Committee.

Adjourned, until 1½ o'clock P. M.

### AFTERNOON SESSION.

Meeting called to order. The President elect, on being conducted to the Chair by Drs. Porter and Knapp, made a few appropriate remarks.

Dr. Benedict, on retiring from the Chair, delivered an appropriate and interesting address.

On motion, it was ordered, that the Address of the retiring President, Dr. Benedict, be published, together with the proceedings of this meeting, in the "Dental Register."

The subject of "Treatment of Exposed Nerves," was then taken up.

Dr. Robinson thinks cobalt better than arsenious acid; it causes less inflammation and pain, removes the nerve in twenty-four hours after the application.

Dr. Porter uses chloride of zinc, in curing ulceration.

The subject of "Diseased Antrums" was next taken up.

Dr. Robinson gave a case of twelve years' standing, which came under his hands, cured by extraction of teeth.

Dr. Benedict is now successfully treating a case with cold water.

Adjourned, to 6½ o'clock P. M.

EVENING SESSION.

Met according to adjournment. Anæsthetic Treatment in Extracting Teeth being before the Convention—

Dr. Whiting spoke against using any anæsthetic agency, unless persons were in sound health. Had seen nitrous oxide, but did not approve of it.

Dr. Hawes had used nitrous oxide a good many times, with excellent success. Had extracted fourteen teeth, by administering the gas, three times; but, since he had seen accounts of fatal cases, had used it with great caution. On the whole, preferred it to chloroform. Had one case of fainting, with nitrous oxide; but did not consider it dangerous.

Dr. Porter said that the profession at Ann Arbor did not approve of nitrous oxide, and discarded it entirely.

Dr. Benedict did not approve of nitrous oxide; and quoted good authority of chemists to prove that any administration might prove injurious to the system, and stimulate latent diseases or create new ones that in time would be fatal.

Dr. Gerry had seen many exhibitions of nitrous oxide; but, on the whole, thought it injurious.

Dr. Knapp had used it in a good many cases, with excellent success; but he believed that the profession should not rush into its use too hastily, as there was no doubt that serious difficulties would arise in many cases, and some might prove fatal.

Dr. Watling said the only question before the Convention was, Whether the Convention would sustain its use, and endorse it. The best dentists had not approved it. And he thought that this Association should not endorse any article that might be even more injurious than chloroform.

Dr. Robinson thought its use would get the dentists in a bad way, make tooth-cobblers of them, and seduce them into extracting teeth that might be saved.

Dr. Gerry did not think chloroform injurious, but perfectly harmless, if given in a horizontal position; but recommends administration with plenty of atmospheric air. Preferred it to all anæsthetic agents.

The subject of Facial Neuralgia was next taken up.

Dr. Porter related a case of a young lady, of nervous temperament, with neuralgia so acute that, raising her hair at the side of the face, the pain would become so intense that fainting ensued. Gave alternately two and a half grains tincture of iron and quinine, and found relief from negative pole galvanic battery,—the pain entirely leaving the patient.

Dr. Watling gave a case of a lady who suffered much from sudden transitions from heat to cold. Cured the patient by applying electricity, and small doses of strychnine.

Dr. Gerry thought that external applications of aconite tincture would almost instantly give relief; and doses of one drop of the tincture, taken internally, five or six times a day.

Dr. Hawes gave a case of fits, caused by the cutting of wisdom-teeth.

Voted, that when we adjourn, we adjourn to meet at Ypsilanti on the fourth Tuesday in January, 1865.

Adjourned, to 9 o'clock to-morrow morning.

WEDNESDAY MORNING SESSION, Jan. 20, 1864.

Meeting called to order, by the President.

Dr. Robinson moved that a committee be appointed to select the individuals, and the subjects for the essays to be read at the next annual meeting; and declared his conviction that it was far better that persons should write upon subjects selected; and if not posted, they should acquaint themselves with the subject, and in that way become more thorough in every department of the profession. Carried, and the following named gentlemen were selected, together with subjects, to-wit:

*Mechanical Dentistry.*—Drs. C. B. Porter, N. A. Gerry, Thomas Rix, and G. H. Mosher.

*Extracting Teeth.*—Drs. J. A. Watling, E. Hawes, H. H. Jackson, and George Field.

*Treatment of Nerves and Alveolar Abscess.*—Drs. C. E. Bartlett, H. Benedict, J. A. Robinson, and G. W. Stone.

*Fitting Artificial Teeth.*—Drs. S. C. Whiting, H. Cole, Wm. Cahoon, and A. F. Barr.

*Dental Education.*—Drs. M. H. Knapp, D. A. Wilder, D. W. Stone, and R. S. Bancroft.

On motion of Dr. Benedict, the Secretary was directed to notify each of the above committees, during the month of July, 1864.

The following resolution, offered by Dr. Robinson, was adopted:

*Resolved,* That a committee of six be appointed, to prepare essays for gratuitous circulation, for the benefit and protection of the profession; and that said committee meet at Ann Arbor, on the day preceding the Spring Commencement, with as many members as may choose to meet at that time; and that the essays be submitted to said committee for criticism and publication. And that every member producing an essay acceptable to said committee, shall be entitled to his proportion of essays when published.

The following named gentlemen were appointed a committee to carry out the above resolution, to-wit: Drs. Whiting, Robinson, Benedict, G. W. Stone, Watling, and Porter.

The Secretary's bill for the preceding year was received, and, on motion, ordered to be paid.

The subject of Filling Teeth was then taken up. Here quite a spirited discussion took place, between Drs. Benedict, Robinson, Stone, and Watling.

Dr. Bartlett read an essay, the subject of which was "The Dental Art, its History and Abuses;" which, on motion, was accepted and ordered to be published.

Adjourned to 1½ o'clock, P. M.

AFTERNOON SESSION.

The afternoon was taken up with clinical exercises.

Dr. Robinson filled the posterior surface of a lower molar, for Dr. Benedict, before the Association.

Dr. Benedict filled the proximal cavity of a superior central incisor, for Dr. Rix.

Also, a crown cavity of a superior molar, for Dr. Rix, by Dr. Stone.

A petition from Dr. Charles Sackrider, of Mason, to become a member of this Association, was received and accepted.

Adjourned, to 6½ o'clock P. M.

EVENING SESSION.

Meeting called to order by the President. The subject of "Hemorrhage from Extraction of Teeth," was taken up for discussion.

Dr. Watling sometimes uses persulphate of iron in cases of hemorrhage.

Dr. Robinson treated a case of hemorrhage of gums with nitrate of silver, using a wash made from white-oak bark, after the hemorrhage had ceased, to heal the gums.

Dr. Watling generally treats constitutionally.

Dr. Whiting stops hemorrhage from extracting, by filling the cavity with sponge.

Dr. Bartlett often uses scraps of sole-leather, placing them in the cavity.

Dr. G. W. Stone uses cobweb and tannin.

Dr. Hawes uses sponge, spirits of camphor, and tannin, covering with a piece of cork compressed.

Dr. Rix compresses linen into the cavity.

Dr. Bartlett knew of a case of death from hemorrhage after the extraction of a tooth.

Dr. Benedict used sponge, in a case of protracted hemorrhage, with success.

On motion, the subject of "Mechanical Dentistry" was next taken up.

Dr. Robinson has no trouble in the springing of rubber plates; scrapes off the top of the impression a little.

Dr. Whiting cuts out the roof of his plates, and makes an air-cell on the alveolar ridge, on each side instead of the center of the plate.

Dr. Robinson thinks there is a great difference in the plaster used; prefers the Grand Rapids plaster to any other.

Dr. Benedict had a case of heating, or fever, of the roof of the mouth, from using a rubber plate; replaced with a gold plate.

Dr. Robinson had heard complaints, two or three times, but did not think them serious. Thinks the rubber is a blessing to the world.

Dr. Benedict has trouble in the breaking of the teeth on rubber plates while in use.

Dr. Palmer saw a dentist who used a lever, ten feet long, with himself on the end, to force his flasks together in packing—man weighed one hundred and eighty pounds—and then complained of cracking of teeth.

Dr. Benedict breaks no teeth in packing.

Dr. Knapp, in packing, weighs his false plate (wax), taking twice that weight of rubber, which will exactly make the plate; thereby having no surplus to crowd against the teeth and break them, and no waste of rubber.

Dr. Benedict gave a case of heating of the mouth by the use of rubber, cured by inserting several gold rivets in the top of the plate, headed on both sides,—the gold being a conductor of electricity.

*Resolved,* That the funds of this Association be appropriated to publishing the essays which shall be contributed and approved by this Convention at its adjourned meeting in Ann Harbor in March next.

Dr. Robinson here made a few remarks, thanking the members for their attendance at this meeting. (See paper.)

On motion, the following named gentlemen were appointed as delegates to the American Dental Association, which meets at Niagara Falls, on Tuesday, July 26th, 1864,—each one being allowed to appoint a substitute, in case he can not be in attendance, to-wit: Drs. J. A. Robinson, S. C. Whiting, C. E. Bartlett, G. W. Stone, G. L. Field, and C. B. Porter.

On motion, the Association adjourned, to meet at Ypsilanti, on the fourth Tuesday in January, 1865, at 9 A. M.

GEO. H. MOSHER, Cor. Sec'y.

---

REMARKS OF DR. J. A. ROBINSON, AT THE CLOSE OF THE CONVENTION.

*Gentlemen of the Association* :—I thank you for your attendance to this Convention; and for the cheer and encouragement you have afforded us by your counsel and deliberations.

When, at the close of your last annual meeting, I invited you, in behalf of the dentists of Jackson, to hold the next Convention in this city, I promised to do all in my power to make your visit agreeable and profitable; and I am happy to hear from so many that their most sanguine expectations have been realized.

Dental Associations should be cherished, on account of their privileges; and they will be prized according to our capacities. When the retiring President remarked that a student of six months' growth had told him a few days ago that treating diseased teeth was a humbug, we all must have felt the necessity of demanding of those about to enter the profession, a more thorough dental education. Make it imperative upon the young dentist that he shall devote three years of study, or graduate at one of our Dental Colleges, and we need not fear for the character of those who call themselves dentists. We prize what we possess, just in propertion to what it has cost us; and education is no exception to this rule. Many excellent operators have given half their lives and strength, in trying to convert the world to the real merits of the profession, and in undoing what some worthless quacks have been trying in vain to do. It is not only important that the *dentist* should be educated; but it is equally so, that the community should be well informed in regard to the real merits of our profession; for without a well-informed clientele, the dentist, however worthy, will languish and starve. The most important means to be used to produce this result, has been almost entirely neglected. We are a reading people; and while we have dental literature enough for our culture and improvement, we should see to it that our patrons have suitable reading on the subject of dentistry, to protect them against any imposters who are always ready to promise to do what they are never able to perform. Let us circulate tracts among the people, to stimulate them to the importance of preserving their teeth, which are so necessary for their health, comfort and good looks; for, next to our lives and liberties, our happiness depends somewhat upon a good set of teeth.

Professional jealousies are always confined to the more ignorant; and education alone will enable us to rise above our catchpenny caterings to the prejudices and jealousies of the uninformed. Associated effort will protect us against any and all the difficulties we may have to encounter, and elevate our calling to a place among the honorable professions of the world.

